# Nephrogenic Adenoma Arising From a Female Urethral Diverticulum: A Case Report and Potential Diagnostic Pitfalls

**DOI:** 10.7759/cureus.36578

**Published:** 2023-03-23

**Authors:** Sarita Thanedar, Joseph M Gosnell, Cecilia G Clement, Eduardo Eyzaguirre, Juan Carlos Alvarez Moreno

**Affiliations:** 1 Pathology, University of Texas Medical Branch at Galveston, Galveston, USA

**Keywords:** skene gland, microcystic urothelial carcinoma, clear cell carcinoma, urethral diverticulum, nephrogenic adenoma

## Abstract

Nephrogenic adenoma is a benign lesion of the urothelial tract characterized by tubules surrounded by thick, hyalinized basement membranes. There is a great variety of architectural patterns within nephrogenic adenomas, including patterns that mimic malignancy, such as focal clear or hobnail cells, areas of significant nuclear atypia, mitosis, and isolated cystic changes. This represents a diagnostic pitfall, where a malignant lesion can be mistaken for a nephrogenic adenoma, leading to a delay in diagnosis and treatment that adversely affects the outcome. In this case report, we describe a nephrogenic adenoma arising in a female urethral diverticulum and discuss the differential diagnosis, which includes clear cell carcinomas, microcystic variant urothelial carcinomas, and Skene’s gland cysts.

## Introduction

The differential diagnosis of vaginal masses can be broad. They may present as simple vaginal wall cysts, urethral diverticulum, or clear cell carcinoma. Nephrogenic adenomas presenting in female urethral diverticula are unusual, with only 30 cases reported in the literature. We present a case of a woman with an anterior vaginal wall lesion diagnosed as a urethral diverticulum with an incidental nephrogenic adenoma and discuss the potential pitfalls in the morphology of this entity in the female genital tract.

## Case presentation

**Figure 1:**
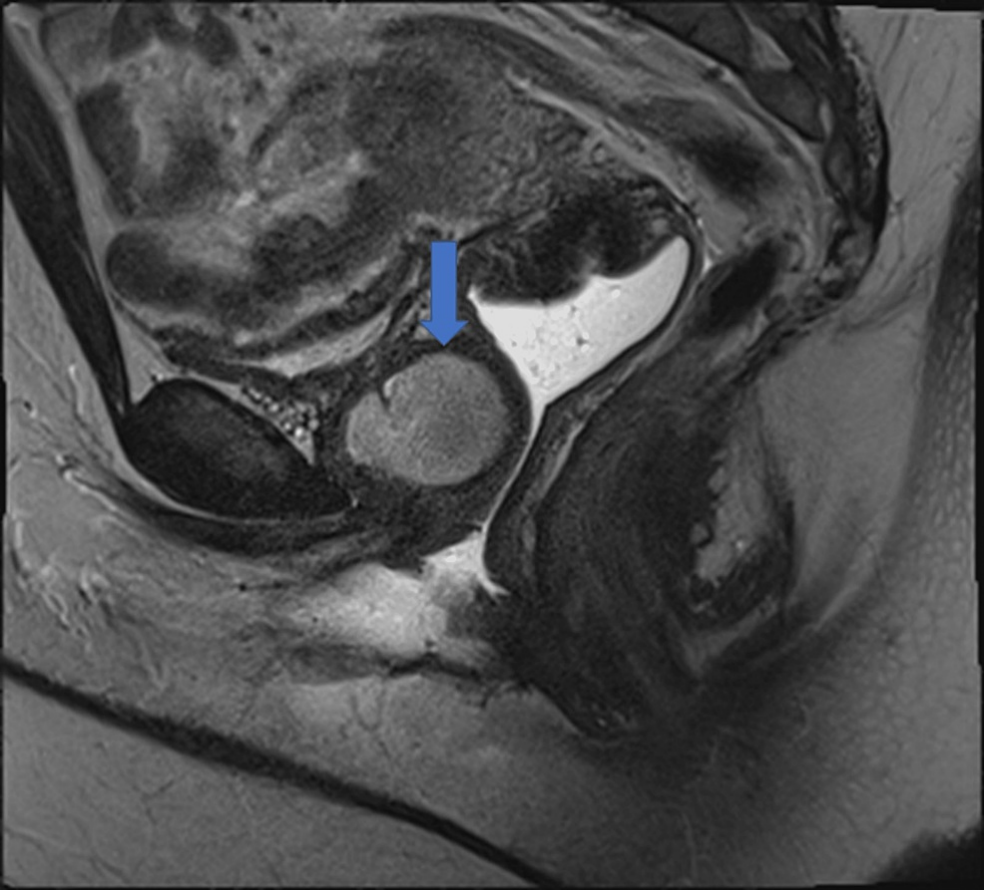
T1 MRI section The image shows a lobulated 3.6 x 2.6 x 3.0 cm lesion (marked with an arrow), likely representing the urethral diverticulum located anterior to the mid-anterior vaginal wall at the level of the pubic symphysis.

A 39-year-old woman was referred from her outpatient clinic to our institution for evaluation of pelvic organ prolapse. Her only medical history was endometriosis. She presented with mixed urinary incontinence, urinary frequency, urgency, dyspareunia, and dysmenorrhea. During her physical exam, a tender mass was appreciated deep in her anterior vaginal wall. An MRI of the pelvis with and without contrast was performed. Results showed a lobulated 3.6 x 2.6 x 3 cm T1 hypointense, T2 mildly hyperintense lesion anterior to the mid-anterior vaginal wall at the level of the pubic symphysis (Figure 1). No communication with the bladder was noted. A thick enhancing wall and internal restricted diffusion were noted to be suspicious of infection. The mass was diagnosed as a likely urethral diverticulum at the time.

The patient underwent a robotic total laparoscopic hysterectomy, bilateral salpingectomy, and urethral diverticulectomy. During her operative procedure, the 3-4 cm bulge was appreciated on her anterior vaginal wall. Drainage yielded purulent fluid. The surgeons backfilled her bladder with methylene blue dye and since no dye was present in the vaginal lesion, the urethral diverticulum was considered unlikely favoring the vaginal cyst which was then partially excised.

**Figure 2:**
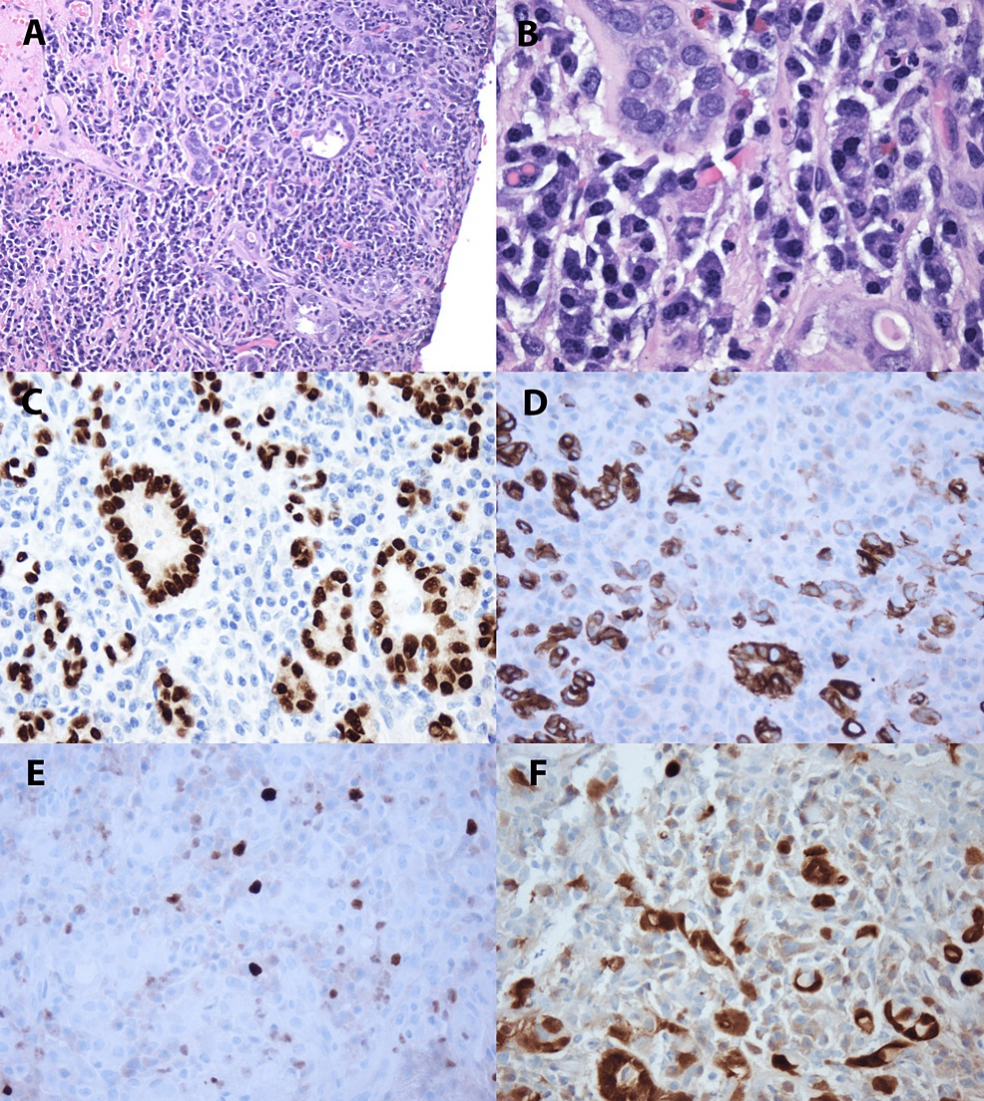
Histopathology images (A) Nephrogenic adenoma with tubular pattern, lined by cuboidal to flattened epithelial cells, in a background of lymphoplasmacytic infiltrate (H&E, 100X). (B) Nephrogenic adenoma with tubules surrounded by hyaline rim; atrophic tubule in lower right (H&E, 400X). (C) PAX-8 and (D) CK7 in Nephrogenic Adenoma (PAX-8 & CK7, 200X). (E) Nephrogenic adenoma with low proliferation index, less than 10%, and (F) patchy positivity with p16 (Ki 67 & p16, 200X).

A gross examination of the cyst demonstrated an irregular fragment of brown hemorrhagic soft tissue measuring 2 cm in the greatest dimension. Microscopic examination showed in a urethral diverticulum a background of fibrosis with severe chronic inflammatory infiltrate and granulation tissue with tubules lined by cuboidal and flat epithelial cells and sparse atrophy, surrounded by a hyaline rim (Figures 2A-2B]. Immunohistochemistry panels showed positive staining for PAX-8 (Figure 2C), CK7 (Figure 2D), and Racemase. Ki 67 shows a low proliferation index (less than 2%) (Figure 2E) and P16 patchy positivity (Figure 2F). CD34, CK20, CDX2, GATA-3, and NKX3.1 were negative. 

At the patient’s two-week post-operative visit, the patient reported improvement in bladder spasms, dysuria, and vaginal pain. She denied urinary frequency, urgency, and incontinence. At the six-week post-operative visit, she was well-healed from her surgery. She reported her urinary urgency and urge incontinence had improved, and she denied dysuria. Her pelvic pain and dyspareunia had also resolved.

## Discussion

Nephrogenic adenomas are benign lesions, derived from renal tubular cells and found anywhere along the urinary tract, most commonly in the bladder [1,2]. Nephrogenic adenomas may present as a palpable mass, rarely at the anterior vaginal wall or in a urethral diverticulum as in our case [3]. Patients with nephrogenic adenoma may have recurrent urinary tract infections (UTIs), obstructive urinary symptoms, hematuria, dyspareunia, or suprapubic pain [3-6].

Most nephrogenic adenomas are small, between 1-2 cm in size. Larger nephrogenic adenomas tend to be 4-7 cm according to the literature [2,3]. On gross inspection, nephrogenic adenomas form papillary lesions but can be polypoid, fungating, or sessile [2]. They can also be sessile, friable, and velvety, mimicking urothelial carcinoma in situ [7]. Nephrogenic adenomas display multiple histologic patterns such as tubules, cysts, papillae, or focal solid growth [2]. The most common feature is tubules surrounded by a thickened hyalinized basement membrane as in our case. They can also present as cystic tubules with colloid-like eosinophilic secretions or mucin within the cysts. The stroma is usually edematous and associated with acute and chronic inflammation. Our case demonstrated a background of granulation tissue which is uncommon. Focal solid areas can have clear cell features, hobnail, cuboidal, or low columnar cells lining the tubules with scant pale cytoplasm. Significant nuclear atypia, mitosis, and deep invasion are not common [2].

Nephrogenic adenomas show diffuse strong nuclear staining for PAX8, S100A1, focal-diffuse staining with BerEP4, and stain negative for P63, PSA, or CEA [8]. These lesions have an excellent prognosis and are treated with removal. If the lesion is in a urethral diverticulum, then diverticulectomy is indicated. With a resolution of symptoms, as in our case [3-7].

The most important entity in the differential diagnosis of nephrogenic adenomas arising in the urethral diverticulum is clear cell carcinoma [7]. Clear cell carcinoma is associated with the urethral diverticula [9]. Patients with clear cell carcinoma in the urinary tract present with hematuria, dysuria, urinary urgency, urinary frequency, and recurrent UTIs, similar to those seen in nephrogenic adenoma [10]. On gross inspection, clear cell carcinomas are solid tumors, commonly invading deeper surrounding structures, such as the muscularis propria.

**Table 1:** Differential diagnosis Pax-8: paired box gene 8; UTI: urinary tract infection; TURBT: transurethral resection of bladder tumor; CK: cytokeratin; PSA: prostatic-specific antigen; CEA: carcinoembryogenic antigen; CA125: cancer antigen 125; CD10: cluster of differentiation 10; MUC: mucin; PRap: Poly (ADP-ribose) polymerase

	Nephrogenic Adenoma	Clear cell carcinoma	Microcystic variant urothelial carcinoma	Skene's gland cyst
Symptoms	Palpable mass, recurrent UTIs, obstructive urinary symptoms, hematuria	Hematuria, dysuria, obstructive urinary symptoms, recurrent UTIs	Hematuria, obstructive urinary symptoms	Asymptomatic, incidental finding
Size	Bimodal: 1-2 cm or 4-7 cm	4cm-6cm	1 cm-6 cm, depending on stage	<1cm
Gross appearance	Papillary lesion without invasion	Solid lesion with invasion	Sessile mass with invasion	No mass present unless in vagina
Clear cells	Only in focal solid areas	Widespread	Common	Rare
Nuclear atypia	Rare	Common	Common	Rare
Mitotic figures	Uncommon	Common	Few	Rare
Histological features	Tubules surrounded by hyalinized basement membrane	Tubulocystic pattern with hobnail and clear cells	Round-oval cystic change within nests of urothelial carcinoma	Glandular and squamous elements in ectocervical stroma
Treatment	Surgical removal	TURBT, cystectomy, chemotherapy	Surgical removal	No treatment necessary
Immunohistochemistry	PAX8, CK903, S100A1, BerEP4, Negative P63, PSA, CEA	Positive CK, CK7, CK20, CA125, CEA; Negative CD10	Positive CK7, CK20, MUC1, MUC5AC, p63, and GATA3	Positive PSA, PRaP

Histologically, clear cell carcinoma displays a tubulocystic pattern, with tubules surrounded by hobnail cells and clear cells predominant throughout the lesion. Nuclear pleomorphism and atypia are common. Variants of clear cell carcinoma with less atypia or fewer cystic areas may also mimic nephrogenic adenomas. Clear cell carcinoma stains positive for CK7, CK20, CA125, CEA, PAX8, and Napsin A, and stain negative for CD10 [10] (Table 1). Histopathologic features that favor clear cell carcinoma over nephrogenic adenoma include a predominance of clear cells, severe cytological atypia, high mitotic rate, necrosis, high Ki-67 proliferative index, and strong staining for p53.

Grossly, both nephrogenic adenomas and microcystic variant urothelial carcinomas appear as sessile masses in the bladder [7,11]. Microcystic variant urothelial carcinoma is an aggressive variant of transitional cell carcinoma of the bladder. Clinically, it presents with gross or microscopic hematuria, and obstructive urinary symptoms [12]. Histologically, this variant presents numerous microcysts and widespread cystic change within nests of urothelial carcinoma (Table 1) [13]. Cysts can be round-oval shaped or slit-like. They may also contain secretions or be calcified. Cysts vary from 1-2 mm long; the larger ones may have flattened epithelium or denuded lining. Infiltration of the muscularis propria is common [11]. Nephrogenic adenomas may have a focal cystic change in their solid areas, but it is not as widespread [2]. Microcystic urothelial carcinoma is treated with surgery, nephrectomy if in the kidney, and a transurethral resection or radical cystectomy if in the bladder. The prognosis is poor, with the invasion of deeper structures being a common manifestation [11].

Skene’s gland cysts may also resemble nephrogenic adenoma histologically [7]. Skene’s glands are the female equivalent of the prostate and are located on either side of the female urethra in their native location but can occasionally be found anywhere along the female genital tract [14]. Misplaced Skene’s gland cysts should be considered in the differential diagnosis of anterior vaginal masses. Basic morphological features of Skene’s gland cyst include glandular and squamous elements in the ectocervical stroma, sometimes forming a double cell layer (Table 1). When found in the lower vagina, these cysts appear as glandular epithelium with focal proliferation, resembling a nephrogenic adenoma [15]. Clinically, a Skene’s gland cyst is silent [15]. It is almost always an incidental finding, secondary to a primary procedure. Skene’s gland cysts have been found during hysterectomies, punch biopsies, or loop excisions, to name a few. The gross appearance differs by location. No mass was present in the cervix or vagina, but Skene’s gland cysts do form a mass when found in the vagina [14]. As Skene’s glands are homologous to the male prostate, they too stain positive for prostate markers. In 13 of 26 cases, Skene’s gland cysts stained positive for PSA (prostate-specific antigen) and stained positive for prostatic acid phosphatase in 16 of 26. Some cysts stained negative for both (six cases) [14].

## Conclusions

Nephrogenic adenomas are benign lesions hypothesized to arise from the urothelium in response to trauma. They have a broad histologic spectrum that can mimic other pathologies leading to difficulties in diagnosis. These mimickers, such as clear cell carcinoma or microcystic urothelial carcinoma, are of worse prognosis.

Clinically, symptoms are often not helpful with diagnosis, with recurrent UTIs, obstructive urinary symptoms, and hematuria being common across the board. Histologically, biopsies can be misleading since nephrogenic adenomas have areas of cystic change and hobnail cells that, when taken out of the context of the whole lesion, can mimic malignant lesions. Immunohistochemical panels have proven to be especially helpful when distinguishing nephrogenic adenomas from the malignant pathologies it mimics.
